# By how much could screening by primary human papillomavirus testing reduce cervical cancer incidence in England?

**DOI:** 10.1177/0969141316654197

**Published:** 2016-06-30

**Authors:** Alejandra Castanon, Rebecca Landy, Peter Sasieni

**Affiliations:** Centre for Cancer Prevention, Wolfson Institute of Preventive Medicine, Barts and The London School of Medicine and Dentistry, Queen Mary, University of London, London, UK

**Keywords:** HPV testing, cervical cancer screening, incidence of cervical cancer, cancer prevention

## Abstract

**Objective:**

The replacement of cytology with human papillomavirus testing as the primary cervical screening test in England is imminent. In light of newly available evidence, we revised our previous estimates of the likely impact of primary human papillomavirus testing on incidence of cervical cancer.

**Method and results:**

Using screening data on women aged 25–64 diagnosed with cervical cancer in England between 1988 and 2012, we previously reported that 38.8% had a negative test six months to six years prior to diagnosis. However, not all of these cancers would be prevented by human papillomavirus testing: for 1.0% the human papillomavirus positive test would come too late (within 18 months of diagnosis) to make a difference; 7.6% will have a negative human papillomavirus test (based on 79.9% sensitivity of human papillomavirus testing in cytology negative women); and 2.0% will develop cancer despite a positive human papillomavirus test. Additionally, we estimate that some women (equivalent to 4.3% of current incidence) whose cancers are currently prevented by cytology-based screening will have a false-negative human papillomavirus test.

**Conclusion:**

Overall, we estimate that 23.9% (95% CI: 19.3–27.6%) of current cases in women invited for screening could be prevented. Based on 2013 cancer incidence statistics, absolute numbers could be reduced by 487 (95% CI 394 to 563) or 3.4 (95% CI 2.8 to 4.0) per 100,000 women per year.

The intention to introduce primary human papillomavirus (HPV) testing in the United Kingdom was recently announced by the UK National Screening Committee.^1^ In 2013, we estimated the impact of introducing HPV primary testing into the English cervical screening programme,^2^ concluding that cervical cancer incidence in women aged 25–64 could be reduced by at most 32.6%, equivalent to 587 cancers (or 4.2 per 100,000 women) per year. Since then Ronco et al.^3^ have published a pooled analysis of randomised controlled trials with cervical cancer as an outcome, comparing HPV testing with cytology. They found that among women with a negative cytology screening test at entry, the rate ratio for invasive cervical carcinoma was 0.30 (95% confidence interval (CI): 0.15–0.60). Here, we update our previous estimates of the impact of primary HPV testing on incidence of cervical cancer in England, in light of this new evidence, and taking into account cancers that will be diagnosed despite a previous positive HPV test.

We used a population-based case–control study of prospectively recorded cervical screening data (from 1988 onwards) for women with cervical cancer (ICD-10 C53) diagnosed aged 25 to 64 in England between April 2007 and March 2012. Data on screening were abstracted from routinely recorded cervical cytology records held on the National Cervical Screening Call/Recall System. After linking, the data were pseudonymised before being transferred to us for analysis. Details of the National Audit of Cervical Cancer have been published previously.^4,5^

Previously, we found that 38.8% (95% CI 37.8% to 39.8%) of women with cervical cancer had a negative cytology test resulting in routine recall (i.e. that did not result in referral to colposcopy or early recall) between six months and six years prior to their diagnosis. Here, we excluded the 1.0% of women whose only negative test within six years of diagnosis was within 18 months of diagnosis, on the grounds that a positive HPV test, together with a negative cytology test, would result in a repeat test a year later, and would probably not have led to the prevention (or even earlier diagnosis) of that cancer.

We modelled the likely results of HPV testing using the following scenario: (i) The overall sensitivity of English cytology to “pre-cancer” (i.e. disease that would lead to a cancer diagnosis over the next six years) is 76.6% (95% CI 65.1–85.1%)^6^; (ii) there will be 70% (95% CI 40–85%) fewer cancers in HPV negative women compared with cytology negative women^3^; (iii) in women who are cytology positive, the sensitivity of HPV to pre-cancer will be 97.0% (95% CI 95.1–98.9%).^7^ These values result in a 93.0% (95% CI 88.0–98.0%) overall sensitivity of HPV testing for identifying women who have or would develop cervical cancer over the next six years, compared with an overall sensitivity of 96.5% (95% CI 93.0–100.0%) in the previous publication (see Table 1).

**Table 1.** Assumed proportions of human papillomavirus (HPV) and cytology test results in women who would develop cervical cancer over the following six years.

	HPV positive, %	HPV negative, %	Overall, %
Cytology positive	74.3 (0.766^a ^ × 0.970^b^)	2.3	76.6^a^
Cytology negative	18.7	4.7	23.4
Overall	93.0	7.0 ((1−0.70^c^) × 0.234)	100.0

a Sensitivity of cytology to CIN2+^6^.

b Proportion of cytology positive women with CIN3 and an HPV positive test result.^7^

c Proportion fewer cancers in HPV negative women compared with cytology negative women.^3^

Of the women with negative cytology from 18 months to 6 years before diagnosis (37.8% of 8774 cases in the audit), 79.9% (95% CI 74.8–85.0%) would have been positive on the HPV test (18.7/23.4, see Table 1), and 7.6% (=37.8% × (1−0.799)) of cancers would be missed.

We further took into account that a number of women will develop (or already have) cancer, despite a positive screen between 18 months and 6 years prior to diagnosis. From Table 1, we estimate that the sensitivity of HPV testing is 1.21 times that of cytology (i.e. 93.0% vs. 76.6%), or 21% (95% CI: 18.1–23.9%) higher. Using the National Audit of Cervical Cancer, we examined the number of cancers diagnosed 18 months to 6 years following an abnormal cytology result (defined as any result which was not negative or inadequate). We identified 833 women with such a test out of a total of 8774 cancer cases (9.5%, 95% CI: 8.9–10.1%). The proportion of cancers despite a positive HPV test (in women with a negative cytology test) due to failures in follow-up or in treatment of CIN would be expected to be an additional 21% of this figure (i.e. of 9.5%), which is 2.0%.

As HPV testing is to become the sole primary screening test in the UK, it is necessary to take into account the proportion of cancers currently prevented by cytology-based screening which would be missed by HPV testing. We estimate this number to be equal to 4.3% of the current total number of cancers (see  Appendix 1, available online).

Taking all of these adjustments into account, we estimate that with HPV primary screening, the number of cervical cancers in women aged 25–64 in England might be reduced by 23.9% (corresponding to 38.8−(1.0 + 7. 6 + 2.0 + 4.3)). Taking into account the uncertainty in the figures combined to reach this percentage, we estimate the 95% confidence interval to be: 19.3% to 27.6% (see Appendix 2 for details, available online).

In our original publication, we estimated a maximum reduction of 587 cancers (or 4.2 per 100,000), using cancer statistics for England^8^ for 2010 to estimate absolute risks. The rate then in women aged 25–64 was 13.0 per 100,000 women, with a total of 1801 cancers. The most recent data are for 2013^9^ show 2039 cancers and a rate of 14.4 per 100,000 women. Applying a reduction of 23.9% to 2013 cancer incidence data yields a reduction of 487 (95% CI 394 to 563) cancers (or 3.4, 95% CI 2.8 to 4.0 per 100,000) in women aged 25–64 by introducing HPV primary testing.

We believe that these updated estimates provide a more realistic estimate of the impact of switching to HPV testing in England. Nevertheless, other factors, such as vaccination (as of September 2015 the first vaccinated catch-up cohorts entered the screening programme) and falling screening coverage (particularly among older women) are not considered here and are likely to have an impact on the effectiveness of HPV primary screening.

## Supplementary Material

Supplementary material

## References

[1] UK NSC recommendations include new bowel cancer screening test [press release]. 15 January 2016, 2016.

[2] Castanon A Landy R Sasieni P How much could primary human papillomavirus testing reduce cervical cancer incidence and morbidity? J Med Screen 2013; 20: 99–103. 24009087 10.1177/0969141313492313

[3] Ronco G Dillner J Elfstrom KM Efficacy of HPV-based screening for prevention of invasive cervical cancer: follow-up of four European randomised controlled trials. Lancet 2014; 383: 524–532. 24192252 10.1016/S0140-6736(13)62218-7

[4] Sasieni P and Castanon A. NHSCSP audit of invasive cervical cancer: National Report 2007–2011, Sheffield, 2012.

[5] NHS Cervical Screening Programme. Audit of invasive cervical cancers. Sheffield, www.gov.uk/government/publications/cervical-screening-auditing-procedures (2006, accessed 2 December 2015).

[6] Cuzick J Szarewski A Cubie H Management of women who test positive for high-risk types of human papillomavirus: the HART study. Lancet 2003; 362: 1871–1876. 14667741 10.1016/S0140-6736(03)14955-0

[7] Kitchener HC Almonte M Thomson C HPV testing in combination with liquid-based cytology in primary cervical screening (ARTISTIC): a randomised controlled trial. Lancet Oncol 2009; 10: 672–682. 19540162 10.1016/S1470-2045(09)70156-1

[8] Office for National Statistics. Cancer statistics registrations, England (Series MB1), No 41, 2012, London: Author, 2014.

[9] Office for National Statistics. Cancer statistics registrations, England (Series MB1), No 44, 2013, London: Author, 2015.

