# Supplementary material for: By how much could screening by primary human papillomavirus testing reduce cervical cancer incidence in England?
Source: J Med Screen. 2016 Jun 30;24(2):110–2. doi: 10.1177/0969141316654197 (PMC5490776; doi:10.1177/0969141316654197)
Supplement: Supplementary material [file MSC654197.pdf]

Appendix 1

We estimate that cytology will have prevented 76.6% of cancers among screened women; with screening coverage of 76.8%: 588 (0.766x0.768x1000) out of 1000 potential cancers would be prevented by cytology, leaving 412 potential cancers. We assume that the sensitivity of HPV testing among women who test positive on cytology is 97.0%. Therefore 3.0% of the 588 (=18) cancers would progress to cancer with HPV testing instead of cytology. This represents 4.3% of the original number of cancers (18/412).

The sensitivity of English cytology to pre-cancer is taken to be 76.6% (95% CI: 65.1%-85.1%), using the sensitivity to CIN2+ in the HART study.[6] The sensitivity of HPV testing to pre-cancer in women with abnormal cytology is taken from the ARTISTIC study[7] based on sensitivity to CIN3+. Combining the revealed and concealed arms, there were 303 women with abnormal cytology and CIN3+ in round 1, of whom 294 (97.0%; 95% CI: 95.1%-98.9%) were HPV positive.

Appendix 2

To calculate a confidence interval for the proportion of cervical cancers prevented by the introduction of HPV primary screening into the NHSCSP programme in England, we assigned a Gaussian distribution to the values used in the calculation (details in Table 2). The full calculation to obtain the 23.9% of extra cancers preventable by HPV primary testing is:

$$B_1-(B_1*(1- B_2))-(B_3* B_4)-((B_5* B_6)*(1- B_7)/(1-(B_5* B_6)))=0.239$$

We randomly generated 10,000 values for each of the point estimates in the formula above, assuming each parameter had a Gaussian distribution with the means and 95% confidence intervals given in Table 2. We combined them to produce 10,000 estimates of the reduction in cervical cancer by introducing HPV primary testing, selecting the 2.5th and 97.5th percentiles to give a 95% confidence interval. This resulted in a 95% confidence interval of 19.3% to 27.6%.

Table 2. Point estimates and range used in the estimation of confidence intervals for the proportion of extra cancers preventable by the introduction of HPV testing in England

	Point estimate	95% CI	Definition
B_1	37.8%	36.8% - 38.8%	Proportion of women in the audit with a negative cytology 18mths to 6yrs prior to diagnosis of cervical cancer (3317/8774).
B_2	79.9%:	74.8% - 85.0%	Proportion of women who are HPV+ given a negative cytology 18mths to 6yrs prior to diagnosis (.187/.234)
B_3	21%	18.1% - 23.9%	Additional women who would develop cancer despite a positive HPV test as a proportion of women who develop cancer despite having positive cytology (0.930/.766 - 1)
B_4	9.5%	8.9% - 10.1%	Proportion of women with cancer in the Audit who develop cancer despite a positive cytology test 18mths to 6yrs prior to diagnosis (833/8,774)
To calculate the 4.3% missed by HPV but prevented by cytology we use:			
B_7	97.0%	95.1% - 98.9%	Proportion of cytology positive women with CIN3 and an HPV positive test result (294/303 from Table 2 in ref (7))
B_5	76.8%	76.2% - 77.4%	Screening coverage observed in the Audit (13323/17341, from Table 1 in ref (2))
B_6	76.6%:	65.1% - 85.1%	Sensitivity of cytology to CIN2+.[6]
